# Sociodemographic factors associated with alcohol consumption among men in Gabon: insights from the Gabon demographic and health survey of 2019–2021

**DOI:** 10.3389/fpubh.2025.1555101

**Published:** 2025-06-09

**Authors:** Gosa Mankelkl, Beletu Kinfe

**Affiliations:** ^1^Department of Biomedical Sciences, College of Medicine and Health Science, Wollo University, Dessie, Ethiopia; ^2^Department of Occupational Health and Safety, College of Medicine and Health Science, Wollo University, Dessie, Ethiopia

**Keywords:** sociodemographic, alcohol, men, prevalence, Gabon

## Abstract

**Background:**

Alcohol is a psychoactive drug that can lead to dependence and has been used extensively for centuries in many different cultures. The primary driver of alcohol consumption is likely its capacity to elevate mood and alleviate stress. More than 200 medical conditions, including liver diseases, traffic accidents, violence, cancer, heart disease, suicide, tuberculosis, and HIV/AIDS, have been causally associated with alcohol consumption. According to estimates from the World Health Organization, 3 million people die from alcohol-related causes globally each year. In other words, 5.3% of all fatalities in the world each year are related to alcohol. Although a majority of research has been conducted in Gabon on this particular subject in a variety of contexts, most of the studies were not representative. Consequently, this study’s primary goal was to evaluate sociodemographic factors associated with alcohol consumption among men in Gabon using data from the most recent national Gabon Demographic and Health Survey.

**Methods:**

For this secondary data analysis, the most recent datasets from the Gabon demographic and health survey were used. A total of 6,894 men participated in this study. The bivariate analysis was used to select the factors for the multivariable analysis. Factors with a *p* < 0.05 significance level were considered significant predictors of alcohol consumption among men in the multivariate analysis. Finally, the 95% confidence intervals for the percentage and odds ratio were reported.

**Results:**

This study includes a total weighted sample of 6,894 men from the Gabon Demographic and Health Survey. The national prevalence of alcohol consumption among men in Gabon was 63.50%, with a 95% confidence interval (CI) of 62.36–64.63. Factors negatively associated with alcohol consumption among men included being above the age of 50 years [AOR: 0.272, 95% CI (0.212, 0.347)], residing in urban areas [AOR: 0.792, 95% CI (0.691, 0.908)], having higher education [AOR: 0.415, 95% CI (0.292, 0.590)], living with a partner [AOR: 0.682, 95% CI (0.570, 0.815)], and listening to the radio [AOR: 0.716, 95% CI (0.632, 0.811)]. In contrast, being a follower of the revival church [AOR: 14.287, 95% CI (11.117, 18.360)] was positively associated with alcohol consumption.

**Conclusion:**

Alcohol consumption among men has been associated with sociodemographic characteristics. Therefore, to reduce alcohol consumption among men, the government of Gabon and other relevant stakeholders should focus on younger men, particularly those living in urban areas and those who have not received formal education. Additionally, media campaigns highlighting the adverse effects of alcohol consumption should be promoted through radio.

## Background

Alcohol is a psychoactive drug that can lead to dependence and has been widely used for centuries across several different cultures (1). The main reason for alcohol consumption is its ability to produce positive emotions and relieve stress (2). Globally, the per capita alcohol consumption increased from 5.5 L in 2005 to 6.4 L in 2016, with an additional increase predicted to reach 7.6 L by 2030 (3).

More than 200 medical conditions, such as liver diseases, traffic accidents, violence, cancer, cardiovascular diseases, suicide, tuberculosis, and HIV/AIDS, have been causally associated with alcohol consumption (4). According to estimates from the World Health Organization, alcohol causes 3 million deaths annually worldwide, accounting for 5.3% of all global fatalities each year (5). Beyond its negative health effects, excessive alcohol consumption also has enormous adverse social and economic impacts on both individuals and society as a whole (6).

In low- and middle-income countries, alcohol consumption is associated with early death and disability (7). The burden of disease and mortality in African nations is significantly influenced by alcohol consumption, and in the upcoming years, alcohol exposure is predicted to increase (8). Furthermore, the alcohol industry is increasingly investing in the African continent as part of a broader plan to boost access, availability, and demand for alcoholic beverages (9).

The effectiveness of alcohol control laws varies widely among African nations. Gabon has some of the fewest laws regulating alcohol consumption in the region (10), which may lead to increased alcohol consumption and related problems (11). In Gabon, drinking is a very common pastime, especially in the countryside, where it is frequently easier to find a bar than a restaurant (12). There are, however, cost-effective policy options available to reduce the harmful use of alcohol. These include pricing regulations, restrictions on marketing and availability of alcohol, better health service responses, and policies and countermeasures against drunk driving (13).

Studies have demonstrated a significant association between alcohol consumption and sociodemographic factors such as age, gender, place of residence, educational level, marital status, and wealth index (14–16). The Gabonese government has endorsed several international agreements and corresponding action plans aimed at enhancing the quality of life and reducing the burden of non-communicable diseases. However, alcohol use received minimal attention, contributing to the increased frequency of non-communicable diseases (17). Although several studies on this specific subject have been conducted in Gabon using various study populations, settings, study periods, and sample sizes, the majority of them lack representativeness. Thus, the main goal of this study was to evaluate the sociodemographic variables associated with alcohol consumption among men in Gabon, using the most recent demographic and health survey data of Gabon. To design and implement effective intervention programs at various levels to decrease alcohol consumption, policymakers require nationally representative data. The results of this study aim to provide stronger evidence for decision-makers and other stakeholders. Additionally, the results can serve as baseline data for future research.

## Materials and methods

### Study setting and period

Gabon is a country on the Atlantic coast of Central Africa, on the equator, bordered by Equatorial Guinea to the northwest, Cameroon to the north, the Republic of the Congo to the east and south, and the Gulf of Guinea to the west. It has an area of 270,000 square kilometers and a population of 2.3 million (18). The Gabon Demographic and Health Survey was implemented by the Direction General of Statistics (DGS) and other stakeholders in collaboration with the Ministry of Health. ICF provided technical assistance for the entire project through the World Demographic Survey Program. Due to the global health crisis caused by coronavirus-2019 (COVID-19), the task of collecting data on the fieldwork was carried out in two phases. The first phase, which took place from 23 November 2019 to 27 March 2020, covered 51% of the total clusters in the investigation sample. The second phase occurred following the easing of restrictive measures to combat COVID-19 in Gabon and covered the period from 7 June to 30 October 2021 (19).

### Data source/data extraction

After permission was secured through an online request by explaining the aim of the study, the data for this analysis were obtained from the Gabon Demographic and Health Survey, which was accessible at the DHS portal.^1^

### Study design

A community-based cross-sectional study design was used. The Gabon Demographic and Health Survey 2019–21 sampling process was designed to ensure representativeness not only at the national level but also at the level of urban and rural areas of residence, as well as the nine administrative provinces of Gabon. The selection procedure for drawing the sample is a random, stratified, and two-stage process. The primary survey unit, also called a cluster, is an enumeration area (EA). In the first stage, 390 clusters were drawn with probability proportional to size by systematic sampling from each province. The size of the cluster is the number of households recorded in the cluster during the general population and housing census. Thirty-two (32) households in the sample were drawn from each selected cluster, making it possible to obtain a list of the households. Then, all men aged 15–64 were eligible in 22 out of 32 households (2 out of 3 households) in each cluster for the men’s survey. In the second stage, all men aged 15–64 who were either permanent residents of the selected households or visitors who stayed in the household the night before the survey were eligible to be interviewed. The men’s questionnaire was used to collect data from 6,894 eligible men (19).

### Study variables

The outcome variable for this study was alcohol consumption. Men who consumed alcohol (almost every day, at least once a week, and less than once a week) were labeled “yes” and coded as “0.” Men who did not consume alcohol at all were labeled “no” and coded “1.”

### Explanatory variables

Age groups, place of residence, educational status, religion, marital status, sex of the household head, wealth index, reading newspapers, listening to the radio, and watching television were considered explanatory variables.

### Data management and analysis

In all analyses, we accounted for the complex nature of the survey design by incorporating clustering, stratification, and weighting. These adjustments were made by dividing the men’s standard weights and their total number in the country by the respective survey sampling fraction. Data extraction, recoding, and both descriptive and analytical analyses were carried out using STATA version 14 software.

A bivariate analysis was conducted to select the factors for multivariate analysis. In the multivariate analysis, variables with a *p* < 0.05 were considered significant predictors of alcohol consumption among men. Finally, frequency, percentage, and odds ratio with a 95% confidence interval were reported.

### Ethical consideration

For this study, an ethical review and participant agreement were not required because the Demographic and Health Survey (DHS) program utilized secondary, publicly accessible survey data. We requested permission from the DHS Program to use the data, which was provided through their website.

## Results

### Sociodemographic characteristics of the participants

A total of 6,894 men participated in this investigation. Among them, approximately 1,170 (16.97%) men aged 15–19 years. A total of 4,562 (66.17%) of the respondents were urban residents; 4,447 (64.51%) were attending secondary education; 2,913 (42.25%) were Catholic; 2,842 (41.22%) were not in union; 5,663 (82.14%) lived in households headed by men; 4,054 (58.80%) were poor; 2,268 (32.90%) read magazines; 3,127 (45.36%) listened to the radio; and 5,579 (80.93%) watched television (Table 1).

**Table 1:** Sociodemographic characteristics of men in Gabon, 2019–2021.

Characteristics (*n* = 6,894)	Categories	Frequency	Percentage
Age groups	15–19	1,170	16.97
	20–24	885	12.84
	25–29	771	11.18
	30–34	814	11.81
	35–39	684	9.92
	40–44	661	9.59
	45–49	583	8.46
	50 and above	1,326	19.23
Place of residence	Urban	4,562	66.17
	Rural	2,332	33.83
Educational status	No education	404	5.86
	Primary	1,276	18.51
	Secondary	4,447	64.51
	Higher	767	11.13
Religion	Catholic	2,913	42.25
	Protestant	1,792	25.99
	Revival church	701	10.17
	No religion	983	14.26
	Other	505	7.33
Marital status	Never in union	2,842	41.22
	Married	1,136	16.48
	Living with a partner	2,139	31.03
	Other	777	11.27
Sex of household head	Male	5,663	82.14
	Female	1,231	17.86
Wealth index	Poor	4,054	58.80
	Middle	1,111	16.12
	Rich	1,729	25.08
Reading newspaper	No	4,626	67.10
	Yes	2,268	32.90
Listening to the radio	No	3,767	54.64
	Yes	3,127	45.36
Watching television	No	1,315	19.07
	Yes	5,579	80.93

### Prevalence of alcohol consumption among men

According to the 2019–2021 Gabon Demographic and Health Survey, the national prevalence of alcohol consumption among men in Gabon was 4,378 (63.50%), with a 95% CI of 62.36–64.63. Among those who consumed alcohol, 1,061 men (15.39%) drank almost every day, 1,596 (23.15%) drank at least once a week, and 1,721 (24.96%) drank less than once a week (Table 2).

**Table 2:** Prevalence of alcohol consumption among men in Gabon in 2019–2021.

Characteristics (*n* = 6,894)	Categories	Frequency	Percentage
Alcohol consumption level	Almost every day	1,061	15.39
	At least once a week	1,596	23.15
	Less than once a week	1,721	24.96
	Not at all	2,516	36.50
Alcohol consumption	Yes	4,378	63.50
	No	2,516	36.50

### Factor analysis associated with alcohol consumption

The results of the bivariable analysis showed that, among men, alcohol consumption was statistically and significantly associated with age groups, place of residence, educational status, religion, marital status, wealth index, reading newspapers, and listening to the radio, and those factors were considered for the multivariable analysis. According to the multivariable analysis, alcohol consumption among men was statistically and significantly associated with age groups, place of residence, educational status, religion, marital status, and listening to the radio.

The finding from this study shows that men aged 50 years and above were 0.272 times less likely to consume alcohol [AOR: 0.272, 95% CI (0.212, 0.347)] compared to those aged 15–19 years. The odds of alcohol consumption among men residing in urban areas were 0.792 times lower [AOR: 0.792, 95% CI (0.691, 0.908)] than among men residing in rural areas. Similarly, men attending higher education were 0.415 times less likely to consume alcohol [AOR: 0.415, 95% CI (0.292, 0.590)] compared to those with no formal education.

Alcohol consumption among men who were followers of the revival church was 14.287 times more likely [AOR: 14.287, 95% CI (11.117, 18.360)] compared to men who were Catholic. The odds of alcohol consumption among men living with partners were 0.682 times lower [AOR: 0.682, 95% CI (0.570, 0.815)] compared to men who had never been in a union. Similarly, men who listened to the radio were 0.716 times less likely to consume alcohol [AOR: 0.716, 95% CI (0.632, 0.811)] than those who did not (Table 3).

**Table 3:** Bivariable and multivariable analyses on factors among men in Gabon 2019–2021.

Characteristics (*n* = 6,894)	Categories	Alcohol consumption		COR with 95% CI; *P*-value	AOR with 95% CI; *P*-value
		Yes	No		
Age groups	15–19	435	735	1	1
	20–24	581	304	0.309 (0.258, 0.371); 0.0001	0.299 (0.247, 0.363); 0.0001
	25–29	552	219	0.234 (0.192, 0.285); 0.0001	0.213 (0.169, 0.268); 0.0001
	30–34	551	263	0.282 (0.234, 0.341); 0.0001	0.253 (0.198, 0.322); 0.0001
	35–39	481	203	0.249 (0.204, 0.305); 0.0001	0.226 (0.174, 0.296); 0.0001
	40–44	452	209	0.273 (0.223, 0.335); 0.0001	0.245 (0.187 0.321); 0.0001
	45–49	404	179	0.262 (0.212, 0.324); 0.0001	0.264 (0.199, 0.349); 0.0001
	50 and above	922	404	0.259 (0.219, 0.306); 0.0001	0.272 (0.212, 0.347); 0.0001
Place of residence	Urban	2,844	1,718	1.161 (1.046, 1.289); 0.005	0.792 (0.691, 0.908); 0.001
	Rural	1,534	798	1	1
Educational status	No education	101	303	1	1
	Primary	788	488	0.206 (0.160, 0.265); 0.0001	0.538 (0.394, 0.736); 0.0001
	Secondary	2,901	1,546	0.178 (0.140, 0.224); 0.0001	0.469 (0.348, 0.632); 0.0001
	Higher	588	179	0.101 (0.076, 0.134); 0.0001	0.415 (0.292, 0.590); 0.0001
Religion	Catholic	2,108	805	1	1
	Protestant	1,082	710	1.718 (1.516, 1.946); 0.0001	1.489 (1.301, 1.705); 0.0001
	Revival church	102	599	15.378 (12.278, 19.260); 0.0001	14.287 (11.117, 18.360); 0.0001
	No religion	723	260	0.941 (0.799, 1.108); 0.471	0.790 (0.662, 0.942); 0.009
	Other	363	142	1.024 (0.830, 1.264); 0.822	1.057 (0.846, 1.320); 0.621
Marital status	Never in union	1,536	1,306	1	1
	Married	605	531	1.032 (0.899, 1.184); 0.652	1.232 (0.985, 1.541); 0.067
	Living with partner	1,634	505	0.363 (0.321, 0.411); 0.0001	0.682 (0.570, 0.815); 0.0001
	Other	603	174	0.339 (0.282, 0.407); 0.0001	0.569 (0.450, 0.721); 0.0001
Sex of household head	Male	3,585	2,078	1.049 (0.922, 1.193); 0.462	
	Female	793	438	1	
Wealth index	Poor	2,627	1,427	1	1
	Middle	655	456	1.281 (1.118, 1.468); 0.0001	1.046 (0.881, 1.241); 0.604
	Rich	1,096	633	1.063 (0.945, 1.195); 0.305	1.056 (0.903, 1.235); 0.488
Reading newspaper	No	2,742	1,884	1	1
	Yes	1,636	632	0.562 (0.504, 0.626); 0.0001	0.880 (0.768, 1.009); 0.068
Listening to radio	No	2,184	1,583	1	1
	Yes	2,194	933	0.586 (0.531, 0.648); 0.0001	0.716 (0.632, 0.811); 0.0001
Watching television	No	826	489	1	
	Yes	3,552	2,027	0.963 (0.851, 1.091); 0.563	

## Discussion

This study demonstrated that the prevalence of alcohol consumption among men in Gabon at the national level was 63.50%, with a 95% CI of 62.36–64.63. This finding was higher than the studies that were conducted in China (45.84%) (20), in India (49.7%) (21), in Ethiopia (46.64%) (22), in Gabon (55.5%) (23), in Ethiopia (24), and in Nigeria (57.9%) (25). The first possible reasons for this variation were a difference in the study period, method of estimation, and sample size. The second possible reason for this variation could be due to the differences in media advertising about alcohol, the availability of alcohol, sociodemographic characteristics, cultural differences, and economic factors, all of which vary with respect to geographic area and may influence drinking behaviors.

According to the multivariable analysis, alcohol consumption among men was statistically and significantly associated with age groups, place of residence, educational status, religion, marital status, and listening to the radio. This finding was concurrent with the studies that demonstrated that alcohol consumption had a significant association with sociodemographic factors such as age, gender, place of residence, educational level, marital status, and wealth index (14–16).

The results of this study indicate that men aged 15–19 had higher odds of consuming alcohol than those aged 50 and above. This finding is supported by the finding of previous research, suggesting that aging leads to the “sick-quitter” effect (26). This effect could be explained by the fact that, as individuals grow older, health problems or prescribed medicines may require them to reduce or completely avoid alcohol consumption. In contrast to the findings of this study, other studies have demonstrated that risky alcohol consumption increases among older age groups (27, 28). It may be due to the fact that health problems or life events such as divorce, loss of family members, or unemployment are common among older people, which may increase the risk of excessive alcohol use.

This result exhibited that men living in urban areas were less likely to consume alcohol than those living in rural areas. This finding is consistent with a study conducted in Gabon (29). People living in rural or remote areas may be influenced by a strong drinking culture, due to stressful issues such as drought and isolation (30). In addition, the availability of locally produced alcohol, social pressure to drink, and cultural norms surrounding alcohol consumption may contribute to the higher rates of alcohol use observed in rural areas. This finding was contrasted by the studies conducted in England and Wales (31), China (32), and Greece (33). These differences may indicate a strong drinking culture where people often use alcohol for getting together with friends, relaxing, and celebrating, and exposure to alcohol-related content on social media is more common in urban areas.

This finding showed that men who attended higher education were less likely to consume alcohol than those who did not attend formal education. This result is supported by studies conducted in Japan (34), in EU countries (35), and in Ethiopia (36). This could be explained by the fact that education enhances cognitive skills that influence decisions that promote health and increases awareness of factual knowledge about health (37–39).

According to this study, men who participated in revival churches were more likely to consume alcohol than those who were Catholic. This difference may be attributed to the interaction between religion and alcohol consumption, which can be influenced by several factors including religious affiliation, level of religiosity, cultural traditions, family influence, geographic locations, and peer networks (40, 41).

This result demonstrated that men living with a partner were less likely to consume alcohol than men who had never been married (42). This finding is supported by a study conducted in Australia (43). This could be explained more by the fact that individuals who never marry appear to exhibit a chronic pattern of heavy alcohol consumption (44).

According to this study, men who listened to the radio were less likely to consume alcohol than men who did not listen to the radio. This may be because listening to the radio stations that broadcast health-related messages can help reduce or prevent alcohol consumption (45). In contrast, other studies have demonstrated that listening to the radio is associated with higher rates of alcohol consumption (46, 47). This association could be better explained by the studies that showed that alcohol-related advertising is associated with high rates of alcohol consumption, cravings, and alcohol addiction. A growing body of research also suggests that these messages may be powerful enough to influence a person’s drinking habits (48–50). Alcohol was frequently portrayed as a drink associated with celebrations and socializing. While responsible drinking messages were provided in 70% of identified alcohol references, these were often vague and ambiguous, for example, by simply stating ‘please drink responsibly’ at the end of an advertisement (46). Furthermore, alcohol advertisements may lead adolescents to have positive attitudes toward alcohol and to start drinking alcohol (51).

### Conclusion and recommendations

The consumption of alcohol among men has been associated with socioeconomic factors. Therefore, to reduce alcohol consumption among men, the government of Gabon and other relevant stakeholders should pay special attention to younger men who live in urban areas and lack formal education. Additionally, they should promote a media campaign via the broadcast channels to inform listeners about the adverse consequences of alcohol consumption.

### Strengths and limitations of this study

The study had many strengths. For instance, the DHS uses a similar design with identical variables in different environments, meaning that the results may be applicable to other similar locations. Additionally, the study employed a sufficiently large sample size at the national level, ensuring its representativeness. However, we would like to highlight a few limitations that should be considered. First, this study was a cross-sectional study, indicating that it does not establish temporal relationships between the independent and dependent variables. The final limitation of this study is related to the community-based design, which depends on participants’ self-reported history of alcohol consumption and does not explain the dose of alcohol consumption.

## Data Availability

Publicly available datasets were analyzed in this study. The data were obtained from the Gabon Demographic and Health Survey 2019–2021, which was found at the DHS portal (https://dhsprogram.com/data/dataset_admin/index.cfm).

## Footnotes

^1^ https://dhsprogram.com/data/dataset_admin/index.cfm
